# Thyroidectomy for Euthyroid Patients with Hashimoto Disease and Persistent Symptoms: An Observational, Postrandomization Study

**DOI:** 10.1155/2024/5518720

**Published:** 2024-04-04

**Authors:** Geir Hoff, Tomm Bernklev, Lene Johnsen, Laurens Reitsma, Dirk Sina, Andromeda Lauzike, Charlotte Gibbs, Tone Hoel Lende, Jon Kristian Narvestad, Rasmus Kildahl, Roald Omdal, Jan Terje Kvaløy, Håvard Søiland

**Affiliations:** ^1^Department of Research, Telemark Hospital, Skien, Norway; ^2^Institute of Clinical Medicine, University of Oslo, Oslo, Norway; ^3^Department of Research, Vestfold Hospital, Tønsberg, Norway; ^4^Department of Breast and Endocrine Surgery, Stavanger University Hospital, Stavanger, Norway; ^5^Department of Breast and Endocrine Surgery, Akershus University Hospital, Campus Oslo, Lørenskog, Norway; ^6^Department of Breast and Endocrine Surgery, Telemark Hospital, Skien, Norway; ^7^Department of Medicine, Telemark Hospital, Skien, Norway; ^8^Department of Gastrointestinal Surgery, Stavanger University Hospital, Stavanger, Norway; ^9^Department of Surgery, Telemark Hospital, Skien, Norway; ^10^Department of Research, Stavanger University Hospital, Stavanger, Norway; ^11^Department of Clinical Science, Faculty of Medicine, University of Bergen, Bergen, Norway; ^12^Department of Mathematics and Physics, University of Stavanger, Stavanger, Norway; ^13^Department of Clinical Science, University of Bergen, Bergen, Norway

## Abstract

**Background:**

Despite adequate hormone substitution in Hashimoto disease, some patients may have persistent symptoms with a possible autoimmune pathophysiology. A recent randomized trial (RCT) using patient-reported outcome measures as the primary endpoint showed benefit in total thyroidectomy, but at a cost of high complication rates.

**Objective:**

To verify results from the RCT in an observational study including a wider range of patients and explore means of predicting who may benefit from such surgery.

**Design:**

A total of 154 patients with Hashimoto disease, euthyroid with or without thyroid hormone substitution, and persistent Hashimoto-related symptoms were subjected to total thyroidectomy and followed for 18 months after surgery. The primary outcome was the General Health (GH) dimensional score in the Short Form-36 Health Survey (SF-36).

**Results:**

Eighteen months after surgery, a clinically significant improvement in GH was seen, similar to the findings in the previous RCT. Anti-TPO antibody titers were markedly reduced after surgery, but preoperative titers or other preoperative parameters could not predict the outcome of surgery. Three (1.9%) of 154 patients experienced permanent unilateral recurrent nerve palsy and six (3.9%) experienced hypoparathyroidism after surgery.

**Conclusions:**

Thyroidectomy had a beneficial symptom-reducing effect in euthyroid patients with Hashimoto disease and persistent symptoms. The pathophysiology of residual symptoms remains unclear, and surgical complication rates are high. If thyroidectomy is considered as a treatment option, it should be performed in dedicated centers with experienced endocrine surgeons and as part of further studies on persistent symptoms. This trial is registered with NCT-02319538.

## 1. Introduction

Hashimoto disease is one of the most prevalent autoimmune disorders worldwide [1]. In addition to gradual destruction of the thyroid gland and a need for thyroid hormone substitution, extra-thyroidal symptoms like chronic fatigue, muscle and joint tenderness, dry mouth and eyes, and poor sleep quality may persist in spite of adequate hormone substitution [2, 3]. These extra-thyroidal symptoms have been difficult to explain [4, 5]. It has been hypothesized that persistent symptoms may be related to extra-thyroidal autoimmune reactions rather than hypothyroidism [6, 7] and that complete removal of antigenic thyroid tissue may attenuate the autoimmune response and relieve symptoms [8, 9]. This hypothesis was supported by the findings in a randomized controlled trial (RCT) on total thyroidectomy published after 18 and 36 months of follow-up [10, 11].

Patients recruited into the above-mentioned RCT could be considered as “end-of-the-road” patients in terms of available treatment options and, therefore, highly motivated for experimental treatment of persistent Hashimoto-related symptoms. A long-lasting placebo effect of surgery could therefore not be excluded [10, 12, 13]. Individuals in the control group were informed that surgery could be an option for them, if results after 18 months of follow-up in the intervention group suggested a beneficial effect of surgery.

In the present study, we tested the reproducibility of the results from the RCT in an observational study of euthyroid patients with Hashimoto disease, persistent symptoms despite being euthyroid with or without thyroid hormone substitution, and with a wider range of anti-TPO antibody levels than in the RCT.

## 2. Methods

**Figure 1:**
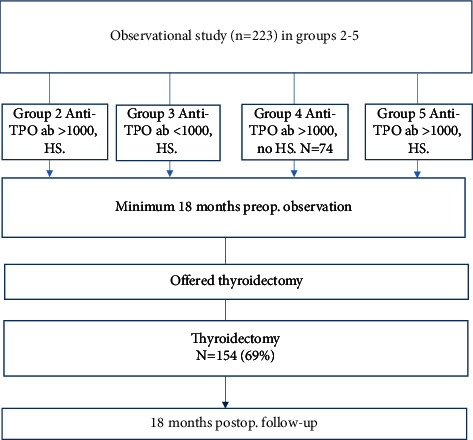
Flow diagram for groups in the observational study. HS = hormone substitution.

Design, methods, and recruitment into the RCT have previously been described in detail [10]. Furthermore, the results from the RCT have been published elsewhere [10, 11]. In brief, this is an open-label trial to which patients were referred by their general practitioner to be considered for inclusion in the RCT, which consisted of 150 patients randomized 1 : 1 to total thyroidectomy or supportive, symptomatic, nonsurgical treatment. Depending on preliminary results 18 months after surgery (intervention group—group 1) in the RCT, a surplus of patients referred (*n* = 148) and the RCT control group (*n* = 75, totally 223 patients) were offered thyroidectomy after at least 18 months of observation of the natural clinical course of the disease (observation study groups 2–5—details in Supplementary material). Similar to the RCT patients, eligible patients for this observational study had persistent Hashimoto disease symptoms despite being euthyroid with or without hormone substitution and elevated levels of antithyroid peroxidase (anti-TPO) antibodies (normal, <100 IU/mL) (Table 1, Figure 1). Typical symptoms reported were fatigue, increased need for sleep with reduced sleep quality, joint and muscle tenderness, and dry mouth and eyes. This report is limited to the observational subsets of patients (*n* = 223 patients).

The primary outcome was the quality of life related to health reported by the patient and measured with the General Health (GH) dimensional score of the generic Short Form-36 Health Survey questionnaire (SF-36). GH was chosen as the most relevant variable to capture subjective health perception assessed directly by the patient. The other SF-36 dimensional scores (bodily pain, mental health, physical functioning, role emotional, role physical, social functioning, and vitality) constituted secondary outcomes together with serum anti-TPO antibody levels. Additionally, total fatigue and the chronic fatigue dimensions were calculated from the Fatigue Questionnaire (FQ) together with a visual analog scale (VAS) with a fatigue score ranging from 0 (“no fatigue”) to 100 mm (“fatigue as bad as it can be”). Safety outcomes such as postoperative recurrent nerve palsy and hypoparathyroidism were monitored. The patients underwent laryngoscopy in the Ear, Nose, and Throat (ENT) department before and after surgery with up to one year of follow-up if there were signs of recurrent nerve palsy. The ENT department was not part of the study team. Postoperative nerve palsy or hypocalcemia that persisted beyond 12 months was classified as permanent adverse events of surgery.

### 2.1. Statistical Analysis

For the primary outcome variable, General Health, we calculated mean scores with 95% CI in each treatment group at each time point. We used paired samples *t*-tests to test for changes in scores from baseline to 18 months after surgery. The same confidence intervals and *t*-tests were calculated for most of the secondary outcome measures. For chronic fatigue, which is dichotomous, we calculated proportions and difference in proportions with 95% CI using the Wilson score method [14], and the McNemar test was used to test for differences from baseline to 18 months. The data for serum anti-TPO antibody titers were highly skewed, and thus, we reported medians and used bootstrapping to calculate CIs.

In the graphs showing changes in patient-reported outcome measures (PROM) over time, we added a line showing age- and sex-adjusted scores representing the average Norwegian background population [15, 16].

To further examine factors associated with improvement in the main outcome variable in the observational groups, we performed logistic regression analyses using as response variable and improvement in GH from the last preoperative measurement to 18 months after surgery of at least 5.3, 8.2, or 9.2 points, respectively, considered to be minimal clinically important difference (MCID) depending on the method used [17, 18]. Sex, age, complications, preoperative GH scores, and anti-TPO antibody levels were included as independent variables.

Data analyses were performed in SPSS version 26 (IBM) and R version 4.2.2 [19].

## 3. Results

Altogether, 223 patients were distributed in the observational study groups as shown in Table 1. Although referred by their GP to be considered for the study, 69 (31%) patients decided not to accept thyroidectomy as a treatment option after 18 months of run-in observation—five of them due to other incidental disease. In one case, surgery was restricted to exploratory when per-operative judgment of the anatomy concluded that thyroidectomy would be too risky. Of the 223 patients, 154 (69%) underwent total thyroidectomy. Ten cases of microcarcinomas (4.5%) were incidental findings during histopathological examination of thyroidectomy specimens.

Surgical complications occurred in 39 (25%) of 154 patients. This included 11 cases of recurrent nerve palsy and 20 cases of hypoparathyroidism. Nine of these were permanent—three cases with unilateral recurrent nerve palsy and six cases with hypocalcemia, altogether a 5.8% risk of permanent unilateral recurrent nerve palsy or hypoparathyroidism.

In a multivariate logistic regression analysis exploring the dependency of the PROM outcome on sex, age, complications, and preoperative values for the anti-TPO antibody and the GH score, a low preoperative GH value was the only variable associated with a clinically significant improvement in the GH score (Table 2). Substituting GH with total fatigue in the model showed similar results (data not shown).

**Figure 2:**
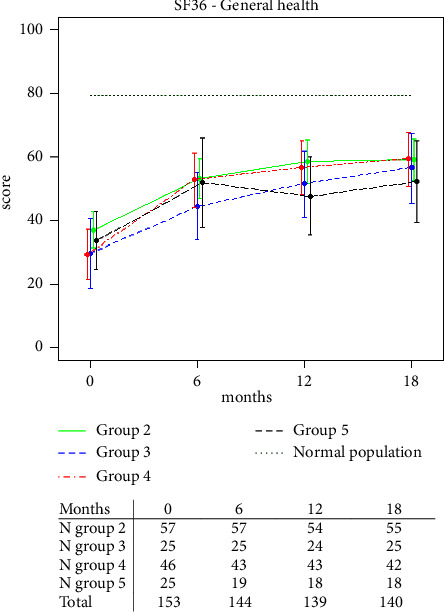
Effects of thyroidectomy on SF-36 General Health score in observational groups 2–5, compared to prevalence scores for age- and sex-adjusted scores considered normal for the Norwegian population. Group 2 is recruited from the RCT control group after serving as controls for a minimum of 18 months prior to being offered surgery. Numbers at risk in the table under the graph.

PROM data were missing for one out of 154 patients who underwent thyroidectomy. The SF-36 General Health score improved during follow-up after thyroidectomy (Figure 2, Supplementary Table 1). Analyses of data in the other SF-36 domains (bodily pain, mental health, physical functioning, role emotional, role physical, social functioning, and vitality) rendered similar results (Supplementary Table 1, Supplementary Figures 1–7). The same applied to total fatigue, chronic fatigue, and fatigue VAS scoring (Supplementary Figures 8–10).

**Figure 3:**
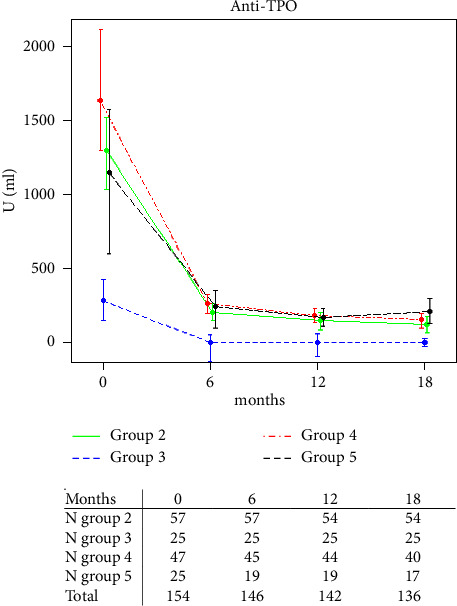
Effect of total thyroidectomy on anti-TPO levels. Numbers at risk in the table under the graph.

Anti-TPO antibody values dropped markedly after surgery (Figure 3). In the multivariate logistic regression model, differences in preoperative anti-TPO antibody levels in the observational groups appeared not to affect the outcome whether using a defined minimal clinical improvement difference (MCID) of 5.3 score points (Table 2), or higher (data not shown for 8.2 or 9.2 score points or a 20% score improvement as thresholds for improvement).

## 4. Discussion

The results already published from our RCT showed that total thyroidectomy has a beneficial effect for patients with Hashimoto disease and anti-TPO antibody level >1000 IU/mL and persistent symptoms despite adequate thyroid hormone substitution for hypothyroidism [10]. In the present observational series of patients, euthyroidism was a requirement for inclusion, whether this was achieved by hormone substitution or the patients had not reached a state of hypothyroidism yet. In contrast to the RCT, patients with elevated anti-TPO antibody values less than 1000 IU/mL were also recruited for the observational study. In the logistic regression analysis with an unlimited range of anti-TPO antibody levels above the normality threshold of 100 IU/mL, preoperative anti-TPO antibody levels did not appear to influence the odds ratio (OR) to obtain a clinically significant improvement in the GH score (Table 2). This is not supportive of autoimmunity being directly responsible for persistent symptoms in patients with euthyroid Hashimoto disease—provided that anti-TPO antibody levels can be accepted as a proxy for general autoimmune activity.

In Hashimoto disease with persistent symptoms, we still lack pathophysiological evidence to support the hypothesis that thyroid tissue serves as an antigen to trigger and maintain immunological reactions against other tissues in addition to the thyroid gland itself. With hindsight, this study may have benefited from a randomized single-blind design with sham surgery in the control group, but the idea was discarded on ethical grounds. A placebo effect lasting 3 years or more as suggested possible in our RCT [11] may be considered less likely in noninvasive drug trials. We do not know, but can only suspect that surgical interventions may have a stronger and longer-lasting placebo effect than nonsurgical treatment [20]. In any case, the estimated duration of placebo effects in drug and vaccine trials cannot be transferred to surgery.

Often in surgery, there is an obvious physical target to handle–a fracture, a tumor, or a deficient organ. A sham surgery control group is easily discarded as unethical. The need to embrace the design of sham surgery studies, however, has been voiced increasingly in recent years, along with higher demands for functional surgery, for example knee and hip surgery in an aging population of patients [12, 13, 21].

When no standard treatment has been shown to be effective for a certain incapacitating condition, this can motivate patients to try a new high-risk invasive method. Total thyroidectomy requires a particularly delicate resection with a high risk of laryngeal nerve palsy or hypoparathyroidism, as shown in the present study. For the majority of patients, however, the relief of having successfully fought surgery without severe and long-lasting complications may contribute to placebo effects far beyond what we see in drug and vaccine trials.

There is some concern that the inflammatory and fibrotic character of Hashimoto thyroiditis may increase the risk of recurrent nerve palsy and hypoparathyroidism. In a prospective observational study including 1268 cases with Hashimoto disease from 68 centers in six European countries, 0.9% had postoperative permanent nerve palsy and 1.1% had permanent hypoparathyroidism (defined as lasting for more than 6 months) [22]. These findings were similar to those seen after surgery for benign goiter: 0.8% nerve palsy and 0.9% hypoparathyroidism [22]. In our study, the number of persistent unilateral nerve palsy was 7 (3.1%), which corresponds to 1.55% per nerve at risk. This is in line with other indications for total thyroidectomy such as thyroid cancer [23, 24]. A more recent report from 90 US hospitals in 11,370 patients subjected to thyroid surgery in 2016–2017 showed that 6.9% of total thyroidectomies caused temporal or permanent laryngeal nerve palsy [25]—very similar to our study (11/154−7.0%). We should bear in mind that the other important laryngeal nerve for the voice function, the superior laryngeal nerve, is not damaged [26]. This nerve supplies the larynx with both efferent motor function (the cricothyroid muscle) and afferent sensory nerve function from the internal larynx area. This nerve may facilitate compensation for a reduced voice function in case of a unilateral recurrent nerve injury.

The purpose of our study was to meticulously remove all thyroid tissue and not leave any potentially immune-stimulating antigenic tissue residues behind. This aim resulted in six (3.9%) cases of permanent hypoparathyroidism after surgery, the symptoms of which were successfully relieved by vitamin D and calcium supplements.

From a PROM perspective and based on the age of a 48-year-old index patient (the average age of the patients in our randomized trial), a US cost-effectiveness study with data from the literature and Medicare cost data concluded that total thyroidectomy is more cost-effective than medical therapy alone for this group of euthyroid patients with Hashimoto disease and persistent symptoms [27]. We ought to be careful not to recommend high-risk surgery using economic arguments as long as a significant placebo effect of the intervention cannot be excluded, although some authors have suggested surgery for selected cases [28]. If total thyroidectomy is considered as an option for selected cases of Hashimoto disease with persistent symptoms, the possible increase in risk of complications is an obvious and major issue. The total thyroidectomy of these cases should be centralized and monitored for quality assurance purposes. An obvious challenge will be how to define selected cases expected to benefit from total thyroidectomy, to be weighed against a high and severe complication rate. Using an improvement in GH score points of 5.3 points as minimal improvement for clinical relevance (MCID) in the present study, neither age or sex nor preoperative anti-TPO antibody levels seem to be of much help (Table 2). As expected, patients with a poor preoperative GH score were more likely to improve after surgery (Table 2), which may be related to the phenomenon of regression to the mean.

As noted above, there are ethical issues to sham operations in randomized trials. Alternatively, there is a risk of endorsing a recommendation for an apparently cost-effective major surgical intervention, which may be no better than a sham operation. This may also be an ethical problem. Total thyroidectomy for Hashimoto disease with persistent symptoms seems presently to be best suited for a randomized, blinded study with sham surgery offered to the control group. A blinded RCT comparing total thyroidectomy with a two-stage hemi-thyroidectomy (e.g., with an 18-month interval) may be a good and more feasible study design than a study relying on a purely and more controversial sham surgery control group.

Hashimoto disease carries an increased risk of carcinoma of the thyroid [29]. Incidental findings of microcarcinomas in the surgical specimen are not unusual. We found microcarcinomas in 10 (6.5%) of 154 thyroidectomy specimens. Another study in 318 patients with Hashimoto disease reported 8% [30]. The role of microcarcinomas has been much debated. So far, recommendations for thyroidectomy based on an increased risk of microcarcinomas among Hashimoto patients have not been made, as patients with these lesions appear to have an excellent prognosis [31, 32].

In conclusion, the present observational study on total thyroidectomy supported findings in a recent RCT that total thyroidectomy may have a beneficial and long-lasting effect for Hashimoto patients with persistent symptoms despite being kept euthyroid with or without hormone substitution. The procedure may be beneficial for selected cases, but the meticulous dissection that is believed to be required comes with a high rate of complications. Anti-TPO antibody titers seem unhelpful in selecting patients for surgery, which should be performed in dedicated centers and as part of further and preferentially blinded studies on the topic of persistent symptoms.

## Figures and Tables

**Table 1:** Group distribution of 223 patients with Hashimoto disease offered thyroidectomy in the observational study.

	Group characteristics		No. of patients		
	Anti-TPO ab (IU/mL)	In need of hormone substitution	Total	Surgery	No surgery
^*∗*^Group 2	>1000	Yes	75	57	18
Group 3	<1000	Yes	34	25	9
Group 4	>1000	^*∗∗*^No	74	47	27
Group 5	>1000	Yes	40	25	15
Total			223	154	69

^*∗*^Controls in the RCT for a minimum of 18 months prior to thyroidectomy. ^*∗∗*^Three patients became in need of hormone substitution during the preoperative period.

**Table 2:** Multivariate logistic regression analysis of collective data from observational groups 2–5 with odds ratios (OR) and 95% confidence intervals to obtain a 5.3 point or higher improvement in General Health (GH) score 18 months after total thyroidectomy.

		GH not improved >5.3 points	GH improved >5.3 points	Univariable OR (95% CI)	Multivariable OR (95% CI)
Sex	Men (%)	3 (20.0)	12 (80.0)	1.0 (reference)	1.0 (reference)
	Women (%)	37 (29.8)	87 (70.2)	0.59 (0.16–2.21), *p*=0.431	0.49 (0.12–2.00), *p*=0.320

Age	Mean (SD)	51.0 (13.7)	46.8 (12.1)	0.97 (0.95–1.00), *p*=0.083	0.97 (0.94–1.01), *p*=0.105

Complications	No (%)	29 (27.9)	75 (72.1)	1.0 (reference)	1.0 (reference)
	Yes (%)	11 (31.4)	24 (68.6)	0.84 (0.37–1.94), *p*=0.689	0.62 (0.24–1.56), *p*=0.305

GH baseline	Mean (SD)	44.7 (25.5)	29.1 (23.4)	0.98 (0.96–0.99), *p*=0.001	0.97 (0.96–0.99), *p*=0.002

Anti-TPO ab baseline	Mean (SD)	1577.4 (1627.2)	2211.0 (2881.0)	1.00 (1.00-1.00), *p*=0.201	1.00 (1.00-1.00), *p*=0.218

## Data Availability

There are restrictions by law in Norway, making data sharing particularly difficult outside Norway.

## Supplementary Materials

Supplementary Materials This contains a detailed description of subgroups in the study and secondary outcome results with graphs.

## References

[1] Caturegli P., De Remigis A., Rose N. R. (2014). Hashimoto thyroiditis: clinical and diagnostic criteria. *Autoimmunity Reviews*.

[2] Ott J., Promberger R., Kober F. (2011). Hashimoto’s thyroiditis affects symptom load and quality of life unrelated to hypothyroidism: a prospective case-control study in women undergoing thyroidectomy for benign goiter. *Thyroid*.

[3] Punzi L., Betterle C. (2004). Chronic autoimmune thyroiditis and rheumatic manifestations. *Joint Bone Spine*.

[4] Jansen H. I., Boelen A., Heijboer A. C., Bruinstroop E., Fliers E. (2023). Hypothyroidism: the difficulty in attributing symptoms to their underlying cause. *Frontiers in Endocrinology*.

[5] Ralli M., Angeletti D., Fiore M. (2020). Hashimoto’s thyroiditis: an update on pathogenic mechanisms, diagnostic protocols, therapeutic strategies, and potential malignant transformation. *Autoimmunity Reviews*.

[6] Saravanan P., Chau W. F., Roberts N., Vedhara K., Greenwood R., Dayan C. M. (2002). Psychological well-being in patients on “adequate” doses of l-thyroxine: results of a large, controlled community-based questionnaire study. *Clinical Endocrinology*.

[7] Groenewegen K. L., Mooij C. F., van Trotsenburg A. P. (2021). van Trotsenburg ASP: persisting symptoms in patients with Hashimoto’s disease despite normal thyroid hormone levels: does thyroid autoimmunity play a role? A systematic review. *Journal of Translational Autoimmunity*.

[8] Chiovato L., Latrofa F., Braverman L. E. (2003). Disappearance of humoral thyroid autoimmunity after complete removal of thyroid antigens. *Annals of Internal Medicine*.

[9] Promberger R., Hermann M., Pallikunnel S. J., Seemann R., Meusel M., Ott J. (2014). Quality of life after thyroid surgery in women with benign euthyroid goiter: influencing factors including Hashimoto’s thyroiditis. *The American Journal of Surgery*.

[10] Guldvog I., Reitsma L. C., Johnsen L. (2019). Thyroidectomy versus medical management for euthyroid patients with Hashimoto disease and persisting symptoms: a randomized trial. *Annals of Internal Medicine*.

[11] Hoff G., Bernklev T., Johnsen L. (2024). Thyroidectomy for euthyroid patients with Hashimoto disease and persisting symptoms. *Annals of Internal Medicine*.

[12] Wartolowska K., Judge A., Hopewell S. (2014). Use of placebo controls in the evaluation of surgery: systematic review. *BMJ*.

[13] Moseley J. B., O’Malley K., Petersen N. J. (2002). A controlled trial of arthroscopic surgery for osteoarthritis of the knee. *New England Journal of Medicine*.

[14] Newcombe R. G. (1998). Improved confidence intervals for the difference between binomial proportions based on paired data. *Statistics in Medicine*.

[15] Loge J. H., Ekeberg O., Kaasa S. (1998). Fatigue in the general Norwegian population: normative data and associations. *Journal of Psychosomatic Research*.

[16] Loge J. H., Kaasa S. (1998). Short form 36 (SF-36) health survey: normative data from the general Norwegian population. *Scandinavian Journal of Public Health*.

[17] Clement N. D., Weir D., Deehan D. (2022). Meaningful values in the Short Form Health Survey-36 after total knee arthroplasty- an alternative to the EuroQol five-dimension index as a measure for health-related quality of life. *Bone and Joint Research*.

[18] Erez G., Selman L., Murtagh F. E. (2016). Measuring health-related quality of life in patients with conservatively managed stage 5 chronic kidney disease: limitations of the Medical Outcomes Study Short Form 36: SF-36. *Quality of Life Research*.

[19] R Core Team (2022). *R: A Language and Environment for Statistical Computing*.

[20] Liu T., Yu C. P. (2011). Placebo analgesia, acupuncture and sham surgery. *Evidence-based Complementary and Alternative Medicine*.

[21] Hetzler P. T., Berger L. E., Huffman S. S. (2023). The characteristics and ethics of sham surgeries: a systematic review of randomized controlled trials. *Annals of Surgery*.

[22] Thomusch O., Sekulla C., Billmann F. (2018). Prospective Evaluation Study of Thyroid Surgery Study G: risk profile analysis and complications after surgery for autoimmune thyroid disease. *British Journal of Surgery*.

[23] Misiolek M., Waler J., Namyslowski G., Kucharzewski M., Podwinski A., Czecior E. (2001). Recurrent laryngeal nerve palsy after thyroid cancer surgery: a laryngological and surgical problem. *European Archives of Oto-Rhino-Laryngology*.

[24] Lo C. Y., Kwok K. F., Yuen P. W. (2000). A prospective evaluation of recurrent laryngeal nerve paralysis during thyroidectomy. *Archives of surgery*.

[25] Gunn A., Oyekunle T., Stang M., Kazaure H., Scheri R. (2020). Recurrent laryngeal nerve injury after thyroid surgery: an analysis of 11,370 patients. *Journal of Surgical Research*.

[26] Soriano R. M., Winters R., Gupta V. *Anatomy, Head and Neck: Larynx Nerves*.

[27] Memeh K., Ruhle B., Vaghaiwalla T., Kaplan E., Keutgen X., Angelos P. (2021). Thyroidectomy for euthyroid patients with Hashimoto thyroiditis and persisting symptoms: a cost-effectiveness analysis. *Surgery*.

[28] Thatipamala P., Noel J. E., Orloff L. (2020). Quality of life after thyroidectomy for Hashimoto disease in patients with persistent symptoms. *Ear, Nose and Throat Journal*.

[29] Xu J., Ding K., Mu L. (2022). Hashimoto’s thyroiditis: A “double-edged Sword” in thyroid carcinoma. *Frontiers in Endocrinology*.

[30] Uhliarova B., Hajtman A. (2018). Hashimoto’s thyroiditis- an independent risk factor for papillary carcinoma. *Brazilian Journal of Otorhinolaryngology*.

[31] Grodski S., Delbridge L. (2009). An update on papillary microcarcinoma. *Current Opinion in Oncology*.

[32] Yeh M. W. (2020). The year in surgical thyroidology. *Thyroid*.

