# Supplementary material for: Thyroidectomy for Euthyroid Patients with Hashimoto Disease and Persistent Symptoms: An Observational, Postrandomization Study
Source: J Thyroid Res. 2024 Apr 4;2024:5518720. doi: 10.1155/2024/5518720 (PMC11008973; doi:10.1155/2024/5518720)
Supplement: Supplementary Materials — This contains a detailed description of subgroups in the study and secondary outcome results with graphs. [file 5518720.f1.pdf]

Supplementary material

Hoff G, et al. Thyroidectomy for euthyroid patients with Hashimoto disease and persisting symptoms.

Definition of patient subsets

Group 1: Intervention group in the RCT. Not included in the present observational study.

Group 2: Control group in the RCT. Recruited with Anti-TPO >1000 IU/mL and euthyroid on hormone substitution. These patients served as controls for a minimum period of 18 months. Patients were then offered thyroidectomy in the same order as they were recruited into the study, and they were followed for another 18 months after surgery. In this group, the patients were considered an observational surgery group from time of surgery onwards.

Group 3: Observational study group recruited with Anti-TPO <1000 IU/mL and euthyroid on hormone substitution. Half-yearly controls for minimum 18 months, i.e., blood samples at preoperative baseline and then at 6, 12 and 18 months after surgery. The last set of data before surgery (PROM- and lab-data) represented patient pre-surgery baseline status.

Group 4: Observational study group recruited with Anti-TPO >1000 IU/mL, but euthyroid without hormone substitution. Half-yearly controls for 18 months before thyroidectomy and 18 months after surgery – similar to group 3.

Group 5: Observational study group recruited with Anti-TPO >1000 IU/mL and euthyroid with hormone substitution, i.e. the same criteria as for inclusion in the randomized trial (groups 1 and 2). Half-yearly controls for 18 months before thyroidectomy and 18 months after surgery – similar to groups 3 and 4. In group 5, five patients were operated without PROM-data follow-up due to hospital capacity problems.

Supplementary table 1. Effect of total thyroidectomy through 18 months of follow-up of the observational groups 2-5 who underwent surgery (n=154), of which 140 patients filled in the SF36 and fatigue questionnaires.

SF36 domains	No. of patients	SF36 score values, mean (95% confidence intervals)				*p-value
		Preop. baseline	6 months postop.	12 months postop.	18 months postop.	
Physical Functioning(PF)	139	61 (57.-65)	74 (71-78)	77. (74-81)	79 (76-83)	<0.001
Role Physical (RP)	140	17 (12-21)	41 (34-48)	48 (41-54)	54 (47-61)	<0.001
Bodily Pain (BP)	138	39 (35-42)	54 (50-58)	56 (52-60)	59 (56-63)	<0.001
General Health (GH)	139	33 (29-37)	51 (47-56)	55 (51-60)	58 (54-62)	<0.001
Vitality (VT)	137	24 (20-27)	42 (38-45)	44 (40-48)	48 (44-52)	<0.001
Social Functioning (SF)	140	42 (37-46)	63 (58-68)	69 (64-73)	70 (65-74)	<0.001
Role Emotional (RE)	140	31 (24-37)	57 (50-64)	63 (57-70)	66 (59-73)	<0.001
Mental Health (MH)	138	56 (52-59)	67 (64-71)	71 (68-75)	72 (69-75)	<0.001
Fatigue						
Total F(score)	140	24 (23-25)	17 (15-18)	16 (14-17)	15. (14-16)	<0.001
**Chronic F	140	79 (73-86)	43 (35-51)	38 (30-46)	31 (23-38)	<0.001
Anti-TPO	136	1271 (1131-1503)	206 (167-247)	147 (123-171)	131 (112-148)	<0.001

*Paired samples *t*-test for scores at baseline and 18 months, McNemar test for Chronic F

**Frequency (%) of cases with chronic Fatigue

Supplementary Fig.1-9. The patient-reported effect of thyroidectomy on SF-36 domains Bodily pain, Mental health, Physical functioning, Role emotional, Role physical, Social functioning and Vitality in subsets of observational series of Hashimoto disease patients (Supplementary Figs 1-7). Similarly, Figs 8-10 show scores for total and chronic fatigue and visual analogue scale (VAS) scores for fatigue.

Supplementary Fig.1. Changes with time after total thyroidectomy for the SF-36 dimension Bodily Pain (BP) in four observational groups (groups 2-5), all compared to a BP score in a normal, mean age- and sex-adjusted Norwegian background population. Numbers at risk in table under the graphs.

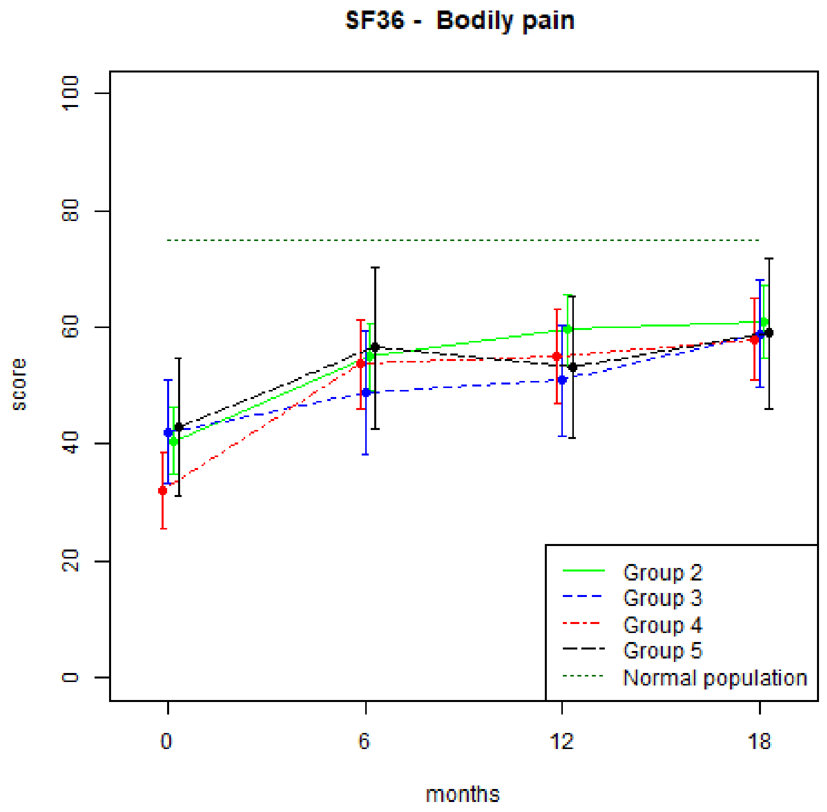

Months	0	6	12	18
N group2	57	57	53	53
N group3	25	25	23	25
N group4	46	44	43	42
N group5	25	20	19	18

Supplementary Fig.2. Changes with time after total thyroidectomy for the SF-36 dimension Mental Health (MH) in four observational groups (groups 2-5), all compared to an MH score in a normal, mean age and sex-adjusted Norwegian background population. Numbers at risk in table under the graphs.

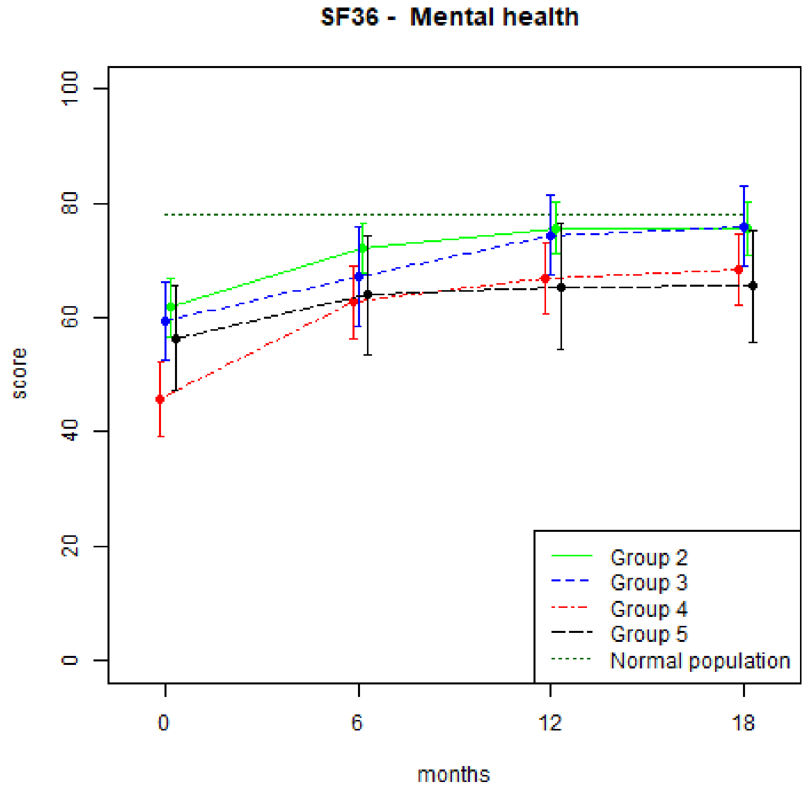

Months	0	6	12	18
N group2	57	57	54	54
N group3	25	25	23	25
N group4	46	44	43	54
N group5	25	20	19	18

Supplementary Fig.3. Changes with time after total thyroidectomy for the SF-36 dimension Physical Functioning (PF) in four observational groups (groups 2-5), all compared to the PF score in a normal, mean age and sex-adjusted Norwegian background population. Numbers at risk in table under the graphs.

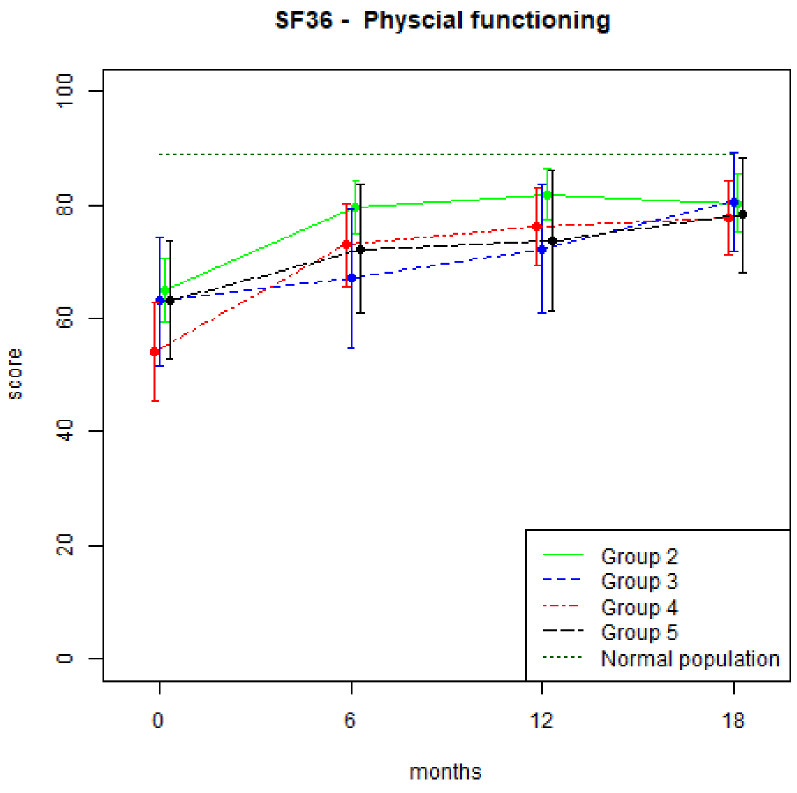

Months	0	6	12	18
N group2	57	55	54	55
N group3	25	24	24	24
N group4	46	44	43	42
N group5	25	20	19	18

Supplementary Fig.4. Changes with time after total thyroidectomy for the SF-36 dimension Role Emotional (RE) in four observational groups (groups 2-5), all compared to the RE score in a normal, mean age and sex-adjusted Norwegian background population. Numbers at risk in table under the graphs.

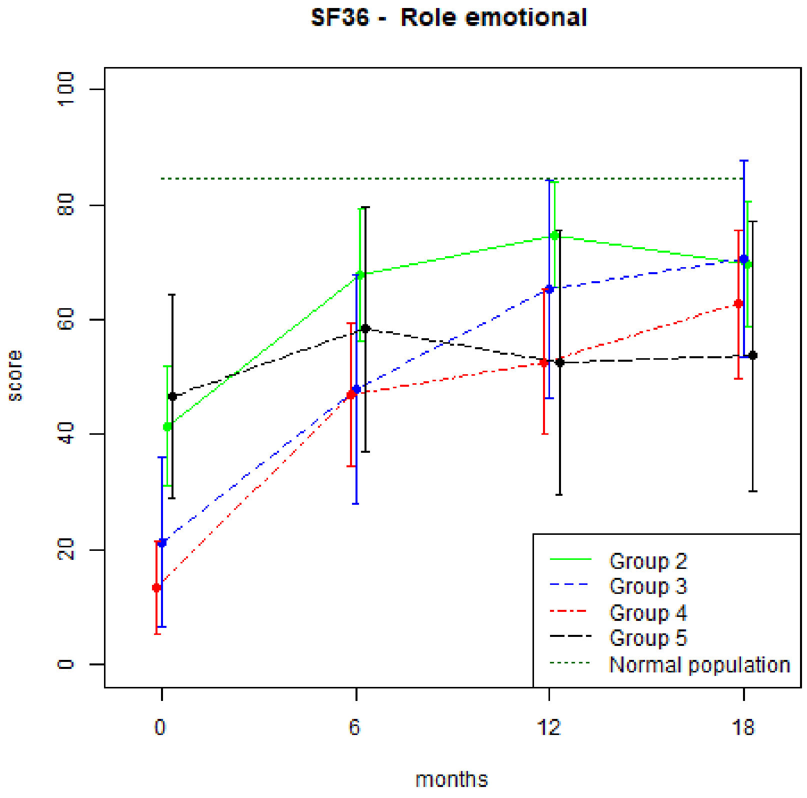

Months	0	6	12	18
N group2	57	57	54	55
N group3	25	25	25	25
N group4	46	44	43	42
N group5	25	20	19	18

Supplementary Fig.5. Changes with time after total thyroidectomy for the SF-36 dimension Role Physical (RP) in four observational groups (groups 2-5), all compared to the RP score in a normal, mean age and sex-adjusted Norwegian background population. Numbers at risk in table under the graphs.

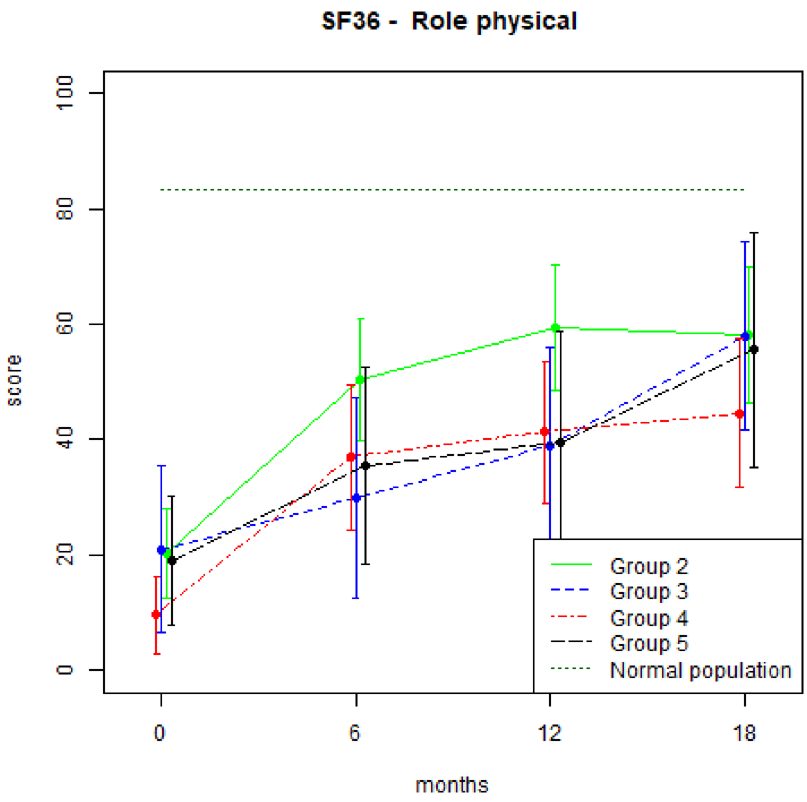

Months	0	6	12	18
N group2	57	57	53	55
N group3	25	25	25	25
N group4	46	44	43	42
N group5	25	19	19	18

Supplementary Fig.6. Changes with time after total thyroidectomy for the SF-36 dimension Social Functioning (SF) in four observational groups (groups 2-5), all compared to the SF score in a normal, mean age and sex-adjusted Norwegian background population. Numbers at risk in table under the graphs.

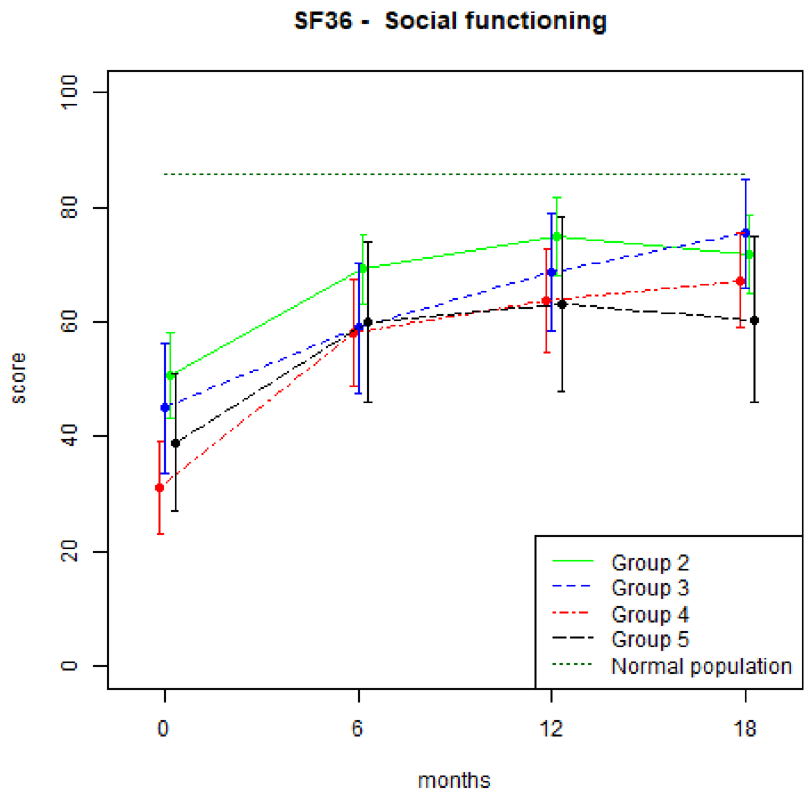

Months	0	6	12	18
N group2	57	57	54	55
N group3	25	25	24	25
N group4	46	44	43	42
N group5	25	20	19	18

Supplementary Fig. 7. Changes with time after total thyroidectomy for the SF-36 dimension Vitality (VT) in four observational groups (groups 2-5), all compared to the VT score in a normal, mean age and sex-adjusted Norwegian background population. Numbers at risk in table under the graphs.

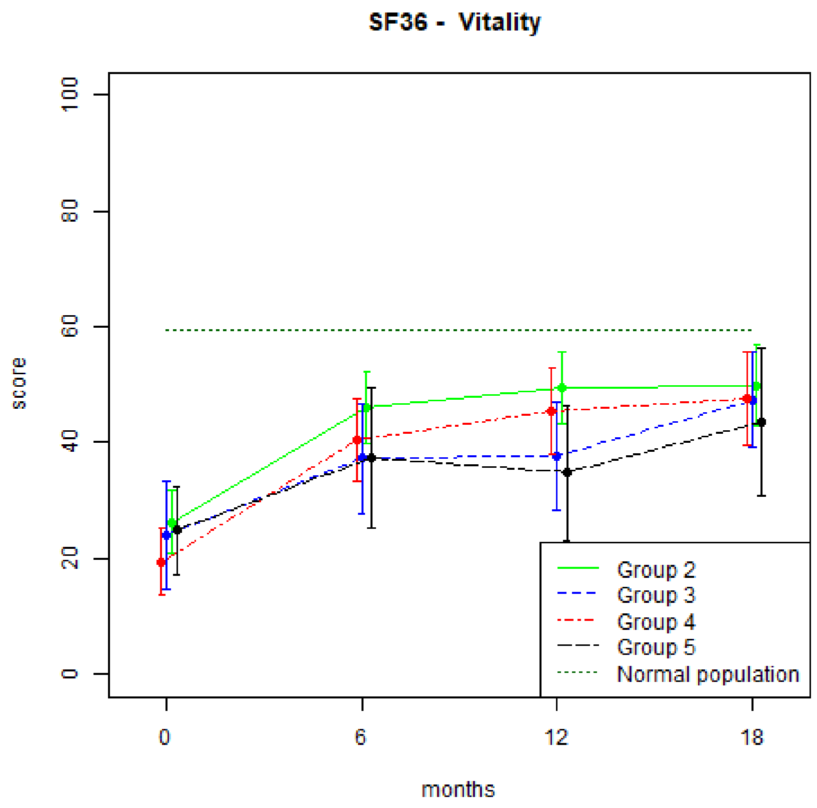

Months	0	6	12	18
N group2	57	57	54	53
N group3	25	25	23	25
N group4	46	44	42	41
N group5	25	20	19	18

Supplementary Fig. 8. Changes with time after total thyroidectomy for total fatigue score in four observational groups (groups 2-5), all compared to the mean total fatigue score in a normal, age and sex-adjusted Norwegian background population. Numbers at risk in table under the graphs.

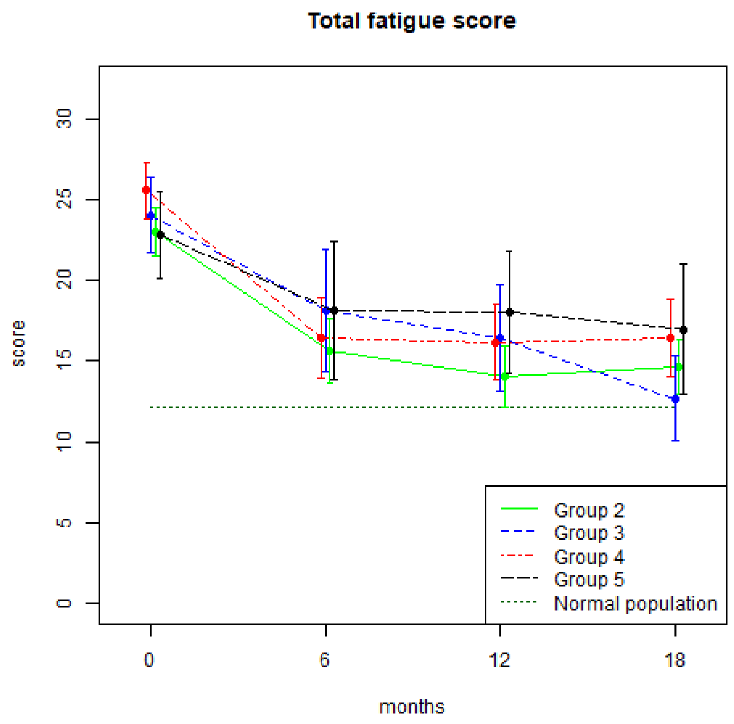

Months	0	6	12	18
N group2	57	57	54	55
N group3	25	25	25	25
N group4	47	44	43	42
N group5	25	20	19	18

Supplementary Fig. 9. Changes with time after total thyroidectomy for chronic fatigue score in four observational groups (groups 2-5), all compared to the mean chronic fatigue score in a normal, age and sex-adjusted Norwegian background population. Numbers at risk in table under the graphs.

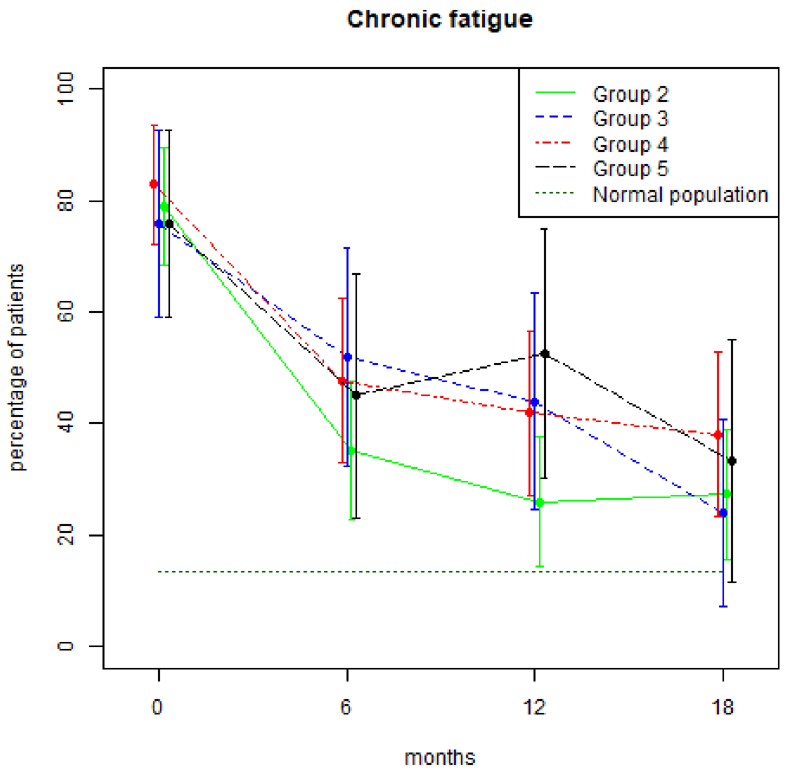

Months	0	6	12	18
N group2	57	57	54	55
N group3	25	25	25	25
N group4	47	44	43	42
N group5	25	20	19	18

Supplementary Fig. 10. Changes with time after total thyroidectomy for Fatigue VAS score in four observational groups (groups 2-5). Numbers at risk in table under the graphs.

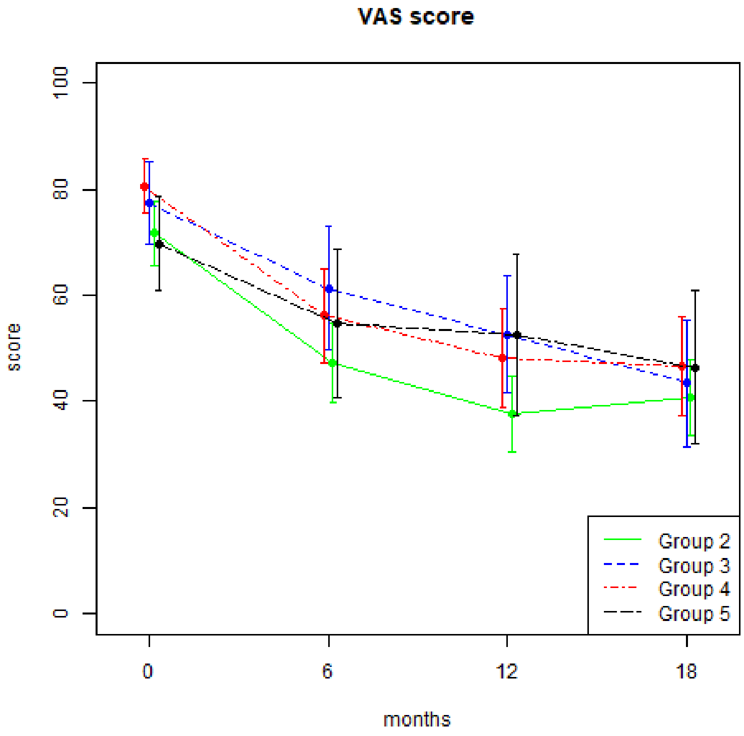

Months	0	6	12	18
N group2	56	57	54	55
N group3	24	25	24	25
N group4	47	44	43	42
N group5	25	19	19	18
